# Integrated Ratio of Metastatic to Examined Lymph Nodes and Number of Metastatic Lymph Nodes into the AJCC Staging System for Colon Cancer

**DOI:** 10.1371/journal.pone.0035021

**Published:** 2012-04-18

**Authors:** Peng Gao, Yong-xi Song, Zhen-ning Wang, Ying-ying Xu, Lin-lin Tong, Jin-liang Zhu, Qing-chao Tang, Hui-mian Xu

**Affiliations:** Department of Surgical Oncology and General Surgery, The First Hospital of China Medical University, Heping District, Shenyang City, People's Republic of China; Baylor University Medical Center, United States of America

## Abstract

**Objective:**

At present, only the number of metastatic lymph nodes (LNs+) is used for the pN category of AJCC TNM system for colon cancer. Recently, the ratio of metastatic to examined lymph nodes (LNR) has been reported to represent powerful independent predictive capacity in colon cancer. We sought to propose a novel category (nLN) which intergrades LNR and LNs+ into the AJCC staging system for colon cancer.

**Design:**

34476 patients from the National Cancer Institute's Surveillance, Epidemiology, and End Results (SEER) dataset with stage III colon cancer were reviewed. Harrell's C statistic was used to evaluate the predictive capacity. The Cox proportional hazards model was used to construct a novel category.

**Results:**

The LNR category had more predictive capacity than the pN category in whole groups of patients (Harrell's C index: 0.6194 vs 0.6113, p = 0.003). Subgroup analysis showed that the LNR category was not better than pN category in predictive capacity if the number of lymph nodes examined was more than 13. We also found that there was significant survival heterogeneity among different pN categories at the same LNR category (P<0.001). The Harrell's C index for our nLN category which intergrades LNR and LNs+ was 0.6228, which was significant higher than that of the pN category (Harrell's C index: 0.6113, P<0.001) or LNR category (Harrell's C index: 0.6194, P = 0.005), respectively.

**Conclusion:**

To evaluate the prognosis of colon cancer, our nLN category which intergrades LNR with LNs+ is more accurate than the pN category or LNR category, respectively.

## Introduction

Colon cancer is one of the most common malignancies [1]. The International American Joint Committee on Cancer (AJCC) TNM staging system is currently regarded as the strongest prognostic parameter for patients with colon cancer [2]. Lymph node metastasis is one of the most important prognostic factors. Determination of the optimal approach to quantifying lymph node status in colon cancer will ensure accurate patient staging, allowing appropriate adjuvant treatment planning and calculation of long-term prognosis.

At present, only the number of metastatic lymph nodes (LNs+) is used for the pN category of AJCC TNM system for colon cancer. This has been criticized as an oversimplification because the number of metastatic lymph nodes is influenced by the total number of examined lymph nodes (eLNs) and may increase the probability of stage migration [3], [4]. As we know, the eLNs pathologically has been demonstrated to affect both staging accuracy and oncological outcomes in node-positive patients [5]. The optimal eLNs for reliable prognostic stratification is less clear until now. According to the guidelines from the AJCC, a minimum of 10–14 lymph nodes must be examined and histopathologically assessed in the tumor specimen to adequately evaluate lymph node status [6]. The college of American Pathologists recommends a minimum of 12 lymph nodes to be examined for colon cancer [7]. Some researchers also proposed that the resection of at least 13, 14 or 15 nodes was associated with prolonged survival in colon cancer for the categories examined [8]–[10]. Unfortunately, surgeons and pathologists do not generally succeed in meeting minimal nodal staging. For those cases without an adequate number of retrieved lymph nodes, the pN category may be not accurate enough.

Over the past few years, the ratio of metastatic to examined lymph nodes (LNR) has been studied widely. Nearly all researchers demonstrated that the LNR is an independent prognostic factor that is highly related to the survival of patients with colon cancer and it has been recommend that the LNR should be applied in prognostic assessment [11]–[18]. However, it is still unclear whether the LNR has more prognostic validity than the AJCC pN category [18], [19].

This study is based on a dataset supported by the Surveillance, Epidemiology, and End Results (SEER) cancer registry with 34476 cases that suffered colon cancer. We found that the LNR category had more predictive capacity than the pN category in the whole groups of patients. However, if the eLNs was more than 13, the LNR category was not better than pN category in predictive capacity. Additionally, there was significant survival heterogeneity among different pN categories at the same LNR category. Finally, we proposed a new category approach that intergraded LNR and LNs+ into the AJCC staging system for colon cancer.

## Materials and Methods

### Data

The dataset we used is the National Cancer Institute's Surveillance, Epidemiology, and End Results (SEER) dataset, 1973–2007. SEER collects data on cancer cases from various locations and sources throughout the United States. Data collection began in 1973 with a limited amount of registries and continues to expand to include even more areas and demographics today. The number of records in the SEER research dataset is up to 6127828 including 5564451 malignant cases. Among these patients, more than 500000 patients suffered from colorectal cancer. Patients with stage III colon cancer diagnosed from 1992 through 2003 were selected for analysis. The primary study endpoint was cancer-specific survival.

Patients were excluded from this study if they had: 1) a prior non-colon cancer or colon cancer other than adenocarcinoma or mucinous adenocarcinomas 2) underwent preoperative radiation, because it was reported that the total number of retrieved lymph nodes may decrease after preoperative chemoradiation [20]; 3) incomplete pathological data entries; or 4) died during the immediate postoperative period (within one month).

After using these exclusion strategies, a dataset consisting of 34476 records was constructed and the following data were recorded: age, gender, race, depth of invasion (determined by SEER's “extent of disease”), histologic grade, number of lymph nodes retrieved, and number of metastatic lymph nodes. Then, the LNR was defined as the ratio of LNs+ divided by the eLNs. To avoid some biases such as the complex category may be over optimized in the comparison of predictive capacity between different categories, models constructed by the categories were found in a training set of data, and then their predictive capacity determined in a test set of data, independent of the training set [21]. Therefore, of the 34476 cases, half were randomly selected for training and the remaining 17238 were used for testing.

### Ethics statement

We have got permission to access the research data file in SEER program.

### Statistical Analysis

Continuous data were presented as the mean ± standard deviation (SD). Cancer-specific survival was analyzed by Kaplan-Meier survival curves, and comparisons were made by the log-rank test. Multivariate analysis was performed using Cox proportional hazards model.

We evaluated the predictive capacity of categories by considering measures of discrimination. Discrimination refers to the ability to distinguish between high-risk and low-risk patients, and was quantified using the Harrell's C statistic, Nagelkerke R^2^, Bayesian Information Criterion (BIC), and time dependent cumulative area under the curve (AUC) [22]–[26]. A model with perfect predictive capacity (sensitivity and specificity of 100%) would have a Harrell's C index of 1.00; a category with higher Harrell's C index was considered more accurate in predictive capacity. The Nagelkerke R^2^ index was also used to score the different categories. R^2^ represents the proportion of variation explained by covariates in regression models. R^2^ is close to 1 for a perfectly predictive model, and close to 0 for a category that does not discriminate between short and long survival times. The BIC was used to assess the overall prognostic performance of different classification systems via bootstrap-resampling analysis. A smaller BIC value indicates a more desirable model for predicting outcome. The AUC was a common tool for the purpose of assessing the predictive power of a continuous variable for a binary outcome and cumulative AUC which was an extension of it to censored survival data was used to evaluate the accuracy of categories in survival prediction at different time.

The cut-off values for sub-groups of LNR were determined using Harrell's C statistic calculated from the training dataset [8], [22]. To study whether the predictive capacity of the LNR category is better than pN category with set standards for the minimal eLNs, a series of tests were performed. There were 20 tests run using a standard for the minimal eLNs from 2 to 21. In each test, a Harrell's C statistic was determined to test the predictive capacity of LNR categories and pN categories.

Comparison of the survival rate among different pN categories stratified by LNR categories was run to analyze the heterogeneity. A log-rank test was run to compare the survival rate among different pN categories in each LNR category.

The novel category (nLN) which combines the pN category with the LNR category is based on the hazard ratio calculated by Cox proportional hazards model. The formula of Cox proportional hazards model is: 
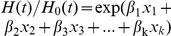
, where 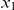
…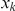 are a collection of predictor variables, LNs+ and LNR in this study, 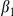
…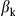 are regression coefficients determined by a least squares approach, and the 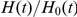 is called the hazard ratio. Moreover, we grouped the calculated hazard ratio four risk levels and formed our nLN category and the optimal cut-off values for the nLN category were also determined using Harrell's C statistic calculated from the training dataset. And then, we compared the predictive capacities of this nLN category with single LNR category and single pN category. Moreover, to test whether the nLN category will have more predictive values irrespective of the eLNs, comparison of the survival rate between patients with <12 eLNs and ≥12 eLNs stratified by all three categories was run.

All the statistical analyses and graphics were performed with the PASW Statistics 18.0 software (SPSS, Inc., Somers, NY, USA), SigmaPlot 12.0 (Systat Software Inc), R version 2.14.0 (The R Foundation for Statistical Computing), Splus 8.0 (Insightful Corporation, Seattle, WA, USA) and STATA MP ver.10 (StataCorp LP, College Station, TX) statistical software. For all analysis, P<0.05 was considered significant.

## Results

According to the 7th edition of the UICC/AJCC TNM staging system, based on the number of positive lymph nodes, patients with different pN categories were divided into: N1a, 34.3% (11826/34476); N1b, 33.8% (11665/34476); N2a, 19.6% (6747/34476); and N2b, 12.3% (4238/34476). Survival differences among the groups were statistically significant (*P*<0.001; Table 1).

**Table 1:** Univariate analysis of the prognostic factors for patients.

	n^a^	5-YSR^b^(%)	*P* *
Sex			0.794
Male	16193	60.4	
Female	18283	60.7	
Age			<0.001
≤60	8991	67.8	
60–75	13745	63.1	
>75	11740	51.8	
Race			<0.001
White	27659	60.6	
Black	3559	57.7	
Other	3258	65.3	
Histologic grade			<0.001
Well	1789	67.7	
Median	23112	63.7	
Poor	9268	52.2	
Undifferentiated	307	49.8	
pT category			<0.001
T1	855	86.4	
T2	2722	80.8	
T3	24328	62.4	
T4a	3871	50.4	
T4b	2700	30.4	
pN category			<0.001
N1a	11826	72.3	
N1b	11665	63.6	
N2a	6747	51.3	
N2b	4238	35.1	
LNR^c^ category			<0.001
LNR1	10492	74.2	
LNR2	7448	66.2	
LNR3	10261	56.6	
LNR4	6275	37.7	
nLN^d^ category			<0.001
nLN1	7747	75.5	
nLN2	12395	67.0	
nLN3	9157	53.6	
nLN4	5177	35.1	

n^a^: Number of patients.

5-YSR^b^: 5-year accumulative survival rate.

LNR^c^: ratio of metastatic to examine lymph nodes.

nLN^d^: the novel category proposed in this study.

* *P* values were made by log-rank test.

Based on optimal cut-off values determined using Harrell's C statistic respectively, patients were divided into the following LNR subgroups: LNR1 = an LNR<0.13; LNR2 = an LNR between 0.13 and 0.24; LNR3 = an LNR between 0.24 and 0.51; and LNR4 = an LNR>0.51. The 5-year survival rate decreased significantly with increasing LNR categories (P<0.001; Table 1).

Moreover, in univariate analysis, age, race, histologic grade and pT categories were also identified as significantly correlated with prognosis (Table 1). In the multivariate analysis, all clinicopathological factors that were significantly correlated with prognosis in univariate analysis were considered. Age, race, histologic grade, pT categories, pN categories, and LNR categories were confirmed to be independent prognostic factors (Table 2). Using Harrell's C statistic to test the predictive capacity of the category in all patients, the LNR categories was significantly better than the pN categories (Harrell's C value: 0.6194 vs 0.6113, respectively, p = 0.003).

**Table 2:** Multivariate Analysis (Cox Proportional Hazard Model) of Prognostic Factors.

	HR^a^	95% CI^b^	*P*
Age	1.022	1.020–1.023	<0.001
Race			<0.001
White*	1		
Black	1.258	1.192–1.328	
Other	0.873	0.822–0.927	
Histologic grade			<0.001
Well*	1		
Median	1.100	1.013–1.195	
Poor	1.316	1.208–1.433	
Undifferentiated	1.442	1.205–1.726	
pT category			<0.001
T1*	1		
T2	1.287	1.063–1.558	
T3	2.538	2.133–3.021	
T4a	3.393	2.838–4.057	
T4b	6.386	5.338–7.640	
pN category			<0.001
N1a*	1		
N1b	1.045	0.988–1.106	
N2a	1.192	1.112–1.277	
N2b	1.479	1.368–1.599	
LNR^c^ category			<0.001
LNR1*	1		
LNR2	1.146	1.060–1.238	
LNR3	1.333	1.219–1.457	
LNR4	1.637	1.459–1.836	
nLN^d^ category			<0.001
nLN1	1		
nLN2	1.170	1.071–1.279	
nLN3	1.312	1.164–1.480	
nLN4	1.646	1.425–1.902	

HR^a^: hazard ratio.

CI^b^: confidence interval.

LNR^c^: ratio of metastatic to examine lymph nodes.

nLN^d^: the novel category proposed in this study.

* : reference category.

**Figure 1:**
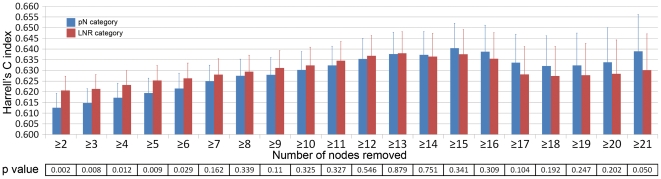
The results of Harrell's C statistical analysis, which reflects the predictive capacity of pN categories and LNR categories using different standards for the minimal number of lymph nodes examined. The p value reflects the significance of comparison between pN categories and LNR categories using different standards for the minimal number of lymph nodes examined.

As seen in Figure 1, following the elevation of standard for the number of minimal eLNs increasing from 2 to 13, the Harrell's C index for LNR category was always higher than that for pN category. The difference between the predictive capacities of the LNR categories and pN categories was significant when the number of minimal eLNs is from 2 to 6 (P<0.05) and the difference lost statistical significance when the number of minimal eLNs was from 7 to 13 (P>0.05). However, as the minimal eLNs increased from 14 to 21, the Harrell's C index for pN category was slightly higher than that for LNR category, but the difference between them was not significant(p>0.05).

**Figure 2:**
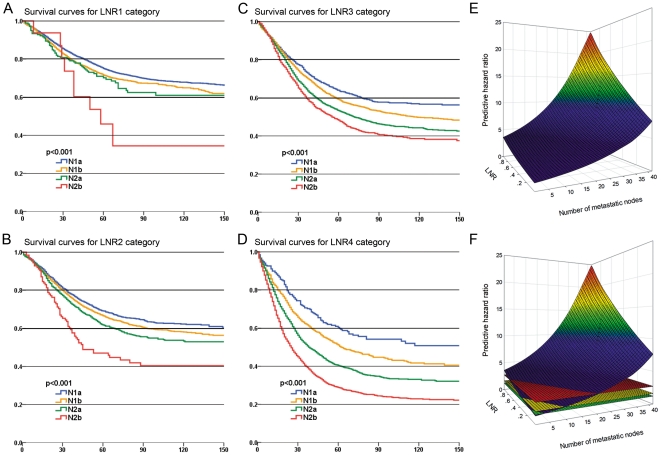
Differences in cause-specific survival in patients of four LNR categories when classified by AJCC pN categories. (**a**) Survival curves for patients of LNR1; (**b**) Survival curves for patients of LNR2; (**c**) Survival curves for patients of LNR3; (**d**) Survival curves for patients of LNR4; (**e**) Mesh plots reflect the predictive hazard ratio based on a Cox proportional hazards model with LNR and LNs+ as covariates. (**f**) Mesh plots with the green, yellow, and red planes which subgroup the hazard ratios into four risk levels (hazard ratio: <1.21, 1.21–1.62, 1.62–1.72 and >1.72).

Using log-rank test, comparison of the survival rates among different LNR categories in different pN categories revealed that there were significant prognostic differences among patients in different pN categories for any LNR category (P<0.001; Fig. 2A, 2B, 2C, 2D). Furthermore, as seen in Figure 2E which reflects the prognostic hazard ratio based on a Cox proportional hazards model with LNR and LNs+ as covariates, at the same LNR level, following the elevation of LNs+, the prognostic hazard ratio increased. That also means there was significant survival heterogeneity among different pN categories at the same LNR category.

A Cox proportional hazards regression with both LNR and LNs+ as covariates was run to calculate the prognostic hazard ratio (HR). After determining the parameters 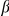, the formula was: 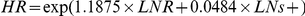. Then, we grouped the patients into four risk levels according to HR and formed the nLN category: nLN1 = an HR<1.21; nLN2 = an HR between 1.21 and 1.62; nLN3 = an HR between 1.62 and 2.72; and nLN4 = an HR>2.72 (Fig. 2F). Patients with different nLN categories were divided into: nLN1, 34.3% (7747/34476); nLN2, 36.0% (12395/34476); nLN3, 26.6% (9157/34476); and nLN4, 15.0% (5177/34476). Survival differences among the groups were statistically significant (P<0.001; Table 1). In the multivariate analysis, the nLN category was significantly correlated with prognosis.

**Figure 3:**
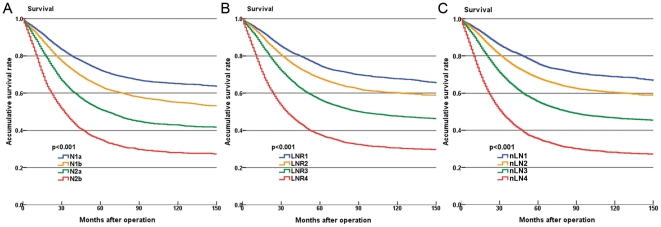
Differences in cause-specific survival in patients of colon cancer when classified by three categories. (**a**) Survival curves for patients classified by AJCC pN categories; (**b**) Survival curves for patients classified by LNR categories; (**c**) Survival curves for patients classified by the novel categories (nLN).

**Figure 4:**
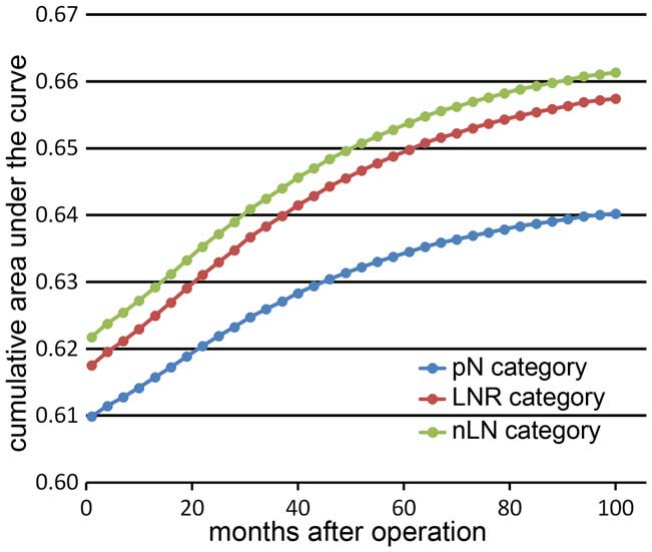
Comparison of cumulative area under the curve analysis among three categories in survival prediction at different time.

Figures 3A, 3B and 3C display the survival curves based on three different category approaches: pN categories, LNR categories and our nLN categories. We compared the Nagelkerke R^2^ and Harrell's C among the three categories. As a result, the nLN category had the highest Nagelkerke R^2^ (pN categories: 0.063; LNR categories: 0.065; nLN categories: 0.072; Table 3). In addition, comparison of Harrell's C statistics and BIC also revealed that our nLN categories had a better predictive capacity than both pN categories and LNR categories (p<0.05; Table 3). Moreover, the results of comparison of cumulative AUC demonstrated that the nLN categories had a higher accuracy in survival prediction than both pN categories and LNR categories at all post-operation time points (Fig. 4).

**Table 3:** Compare Nagelkerke R^2^, Harrell's C and Bayesian Information Criterion among three categories.

	Nagelkerke R^2^	Harrell's C			BIC^a^	
		Coefficient	95% CI^b^	*P* ^c^	Coefficient	*P* ^c^
pN category	0.063	0.6113	0.6046–0.6180	<0.001	−2175.87	<0.001
LNR^d^ category	0.065	0.6194	0.6127–0.6260	0.005	−2404.12	<0.001
nLN^e^ category	0.072	0.6228	0.6162–0.6294		−2581.11	

BIC^a^: Bayesian Information Criterion.

CI^b^: confidence interval.

*P* ^c^: comparing the predictive power of survival models with novel category.

LNR^d^: ratio of metastatic to examine lymph nodes.

nLN^e^: the novel category proposed in this study.

**Figure 5:**
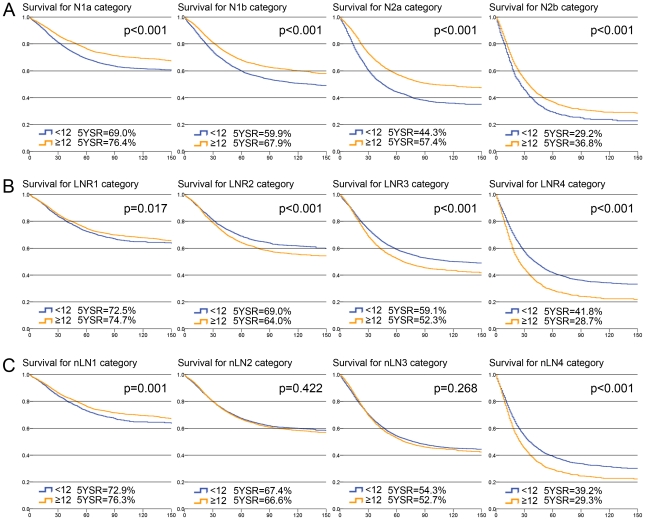
Comparison of of the survival rate between patients with <12 number of examined lymph nodes (eLNs) and ≥12 eLNs stratified by three categories. (**a**) Survival curves stratified by AJCC pN categories; (**b**) Survival curves stratified by LNR categories; (**c**) Survival curves stratified by the novel categories (nLN). The blue lines represent the survival curves of patients with <12 eLNs and the yellow lines represent the survival curves of patients with ≥12 eLNs. The 5-year accumulative survival rates (5YSR) for patients were also presented.

Comparison of the survival rate between patients with ≥12 eLNs and <12 eLNs stratified by all three categories revealed that the heterogeneities of prognosis between patients with <12 eLNs and ≥12 eLNs at nLN categories was the lowest among three categories. In four pN categories there were significant survival differences between patients with <12 eLNs and ≥12 eLNs (5-year accumulative survival rates: 69.0% vs. 76.4% at N1a, p<0.001; 59.9% vs. 67.9% at N1b, p<0.001; 44.3% vs. 57.4% at N2a, p<0.001; 29.2% vs. 36.8% in N2b, p<0.001; Fig. 5A). Although the heterogeneities of prognosis between patients with <12 eLNs and ≥12 eLNs at LNR categories were lower than that of pN categories, there were significant survival heterogeneities in four LNR categories (5-year accumulative survival rates: 72.5% vs. 74.7% at LNR1, p = 0.017; 69.0% vs. 64.0% at LNR2, p<0.001; 59.1% vs. 52.3% at LNR3, p<0.001; 41.8% vs. 28.7% at LNR4, p<0.001; Fig. 5B). Conversely, there were no significant survival heterogeneities between patients with <12 eLNs and ≥12 eLNs at nLN2 and nLN3 (5-year accumulative survival rates: 67.4% vs. 66.6% at nLN2, p = 0.422; 54.3% vs. 52.7% at nLN3, p = 0.268). There were survival differences in nLN1 and nLN4 (5-year accumulative survival rates: 72.9% vs. 76.3% at nLN1, p = 0.001; 39.2% vs. 29.3% at nLN4, p<0.001; Fig. 5C).

## Discussion

Over the past few years, LNR has been studied widely in the prognostic analysis of colon cancer. Nearly all researchers demonstrated that the LNR is an independent prognostic factor. However, it is still unclear whether the LNR category has more prognostic validity than the AJCC pN category [18], [19]. In our study, we compared the predictive capacity of the LNR category with that of pN based on the SEER dataset. We found that the LNR category was significantly better than the pN category in predictive value in whole groups of patients (Harrell's C index: 0.6194 vs 0.6113, respectively, p = 0.003). This result was similar to previous studies [14], [15], [17], [26], [27].

However, there is still debate on whether the LNR has more prognostic validity than the AJCC pN category if the eLNs is enough. Priolli et al. analyzed the prognostic value of the LNR in patients with no less than 12 eLNs and multivariate analysis showed that both the LNR and lymph node involvement were independent prognostic factors. Furthermore, lymph node involvement obtained a higher ‘score’ than the LNR [16]. Recently, based on the SEER dataset, Chen et al. compared the prognostic values of the LNR categories with that of the pN categories in patients with no less than 12 eLNs. Multivariate analysis showed that both LNR and lymph node involvement were independent prognostic factors. They proposed that the LNR categories had better prognostic value than the pN categories for the reason that the LNR categories had a higher hazard ratio than the pN categories [28]. In this study, we found that when the minimal eLNs was no less than 14, the predictive capacity of the pN categories was even higher than the LNR categories, although the difference is not significant statistically. That meant that LNR category was not superior to pN category in the predictive captivity all the time. Maybe this result could be affected by the cut-off value of the LNR categories, while the optimal cut-off value for LNRs has not received consensus [29] and the cut-off values used in this study were searched by statistic method to ensure the efficiency.

Moreover, it was verified that there was significant survival heterogeneity among different pN categories at the same LNR category. Using log-rank test, comparison of the survival rates among different LNR categories in different pN categories revealed that there were significant prognostic differences among patients in different pN categories for any LNR category (P<0.001; Fig. 2A, 2B, 2C, 2D). Therefore, it is not scientific enough if the pN category is simply replaced by LNR category. The result of Cox proportional hazards model with LNR and LNs+ as covariates also supported this opinion (Fig. 2E). Nevertheless, the prognostic value of LNR could not be ignored. Maybe a category which integrated the LNR with LNs+ is considerable.

In light of these considerations, a Cox proportional hazards regression with both LNR and LNs+ as covariates was run to calculate the prognostic hazard ratio (HR). After calculation of the parameters, the formula: 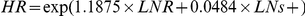 was obtained. Both the LNR and LNs+ were referred by this formula and the large dataset used in this study make sure that the parameters 1.1875 and 0.0484 were accurate. And then, we divided calculated HR into four risk levels and formed our new category (nLN): nLN1 = an HR<1.21; nLN2 = an HR between 1.21 and 1.62; nLN3 = an HR between 1.62 and 2.72; and nLN4 = an HR>2.72.

Survival differences among the groups were statistically significant (P<0.001). Furthermore, using three statistical methods; i.e., Nagelkerke R^2^, Harrell's C and BIC, we verified the effectiveness of the nLN category and compared it with the LNR categories and pN categories, respectively. We found that the nLN category had higher predictive capacity than the other two categories (Table 3). Moreover, based on comparison of cumulative AUC, we found that the nLN categories had a higher accuracy in survival prediction than both pN categories and LNR categories at all post-operation time points (Fig. 4). Furthermore, compared with pN and LNR categories, the nLN category had more value in reduction of the heterogeneity of prognosis caused by insufficient eLNs. In the present study, we found significant heterogeneities of prognosis between patients with <12 eLNs and ≥12 eLNs at all four pN and LNR categories (Fig. 5A, 5B). Conversely, there was no heterogeneities of prognosis between patients with <12 eLNs and ≥12 eLNs at nLN2 and nLN3 of the nLN category (Fig. 5C). To some degree, the nLN category can be used to counterbalance incomplete nodal assessment for pathological evaluation and increase the accuracy of prognostic predication irrespective of the eLNs. These findings indicated that nLN categories were suitable for predicting the prognosis of patients with colon cancer. And then, based on the nLN category, the patients may obtain some clinical benefit from accurate prediction of long-term prognosis and appropriate adjuvant treatment planning.

Our study has some limitations. It is a retrospective exploratory study based on SEER data. Clinical and pathologic patient information can be heterogeneous since SEER collects information from 12 population-based cancer registries. On the other hand, data on adjuvant therapy is limited to information on radiation therapy only and it was reported that the total number of retrieved lymph nodes may decrease after preoperative chemotherapy [20]. Also, there is a lack of information on other factors that are related to the total number of retrieved lymph nodes, such as BMI index [28]. This makes some subgroup analysis impossible. Further, external validation by using other sources of data with sufficient pathological information is needed.

We conclude that, to evaluate the prognosis of colon cancer, our nLN category which intergrades LNR with LNs+ is more accurate than the pN category or LNR category, respectively.
